# Benchtop Zone Refinement of Simulated Future Spent Nuclear Fuel Pyroprocessing Waste

**DOI:** 10.3390/ma17081781

**Published:** 2024-04-12

**Authors:** Alex Scrimshire, Daniel J. Backhouse, Wei Deng, Colleen Mann, Mark D. Ogden, Clint A. Sharrad, Mike T. Harrison, Donna McKendrick, Paul A. Bingham

**Affiliations:** 1 Materials and Engineering Research Institute, Sheffield Hallam University, Sheffield S1 1WB, UK; a.scrimshire@shu.ac.uk (A.S.); daniel.backhouse@glass-futures.org (D.J.B.); wei.deng@shu.ac.uk (W.D.); 2 The Henry Royce Institute and Department of Materials Science and Engineering, The University of Sheffield, Sheffield S1 3JD, UK; 3 Department of Chemistry, University of Sheffield, Western Bank, Sheffield S10 2TN, UK; 4 School of Engineering, The University of Manchester, Oxford Road, Manchester M13 9PL, UK; 5 National Nuclear Laboratory, Sellafield, Seascale, Cumbria CA20 1PG, UK; mike.t.harrison@uknnl.com (M.T.H.);

**Keywords:** pyroprocessing, molten salt, nuclear fuel cycle, LKE, chloride salts, zone refinement

## Abstract

The UK’s adoption of pyroprocessing of spent nuclear fuel as an alternative to the current aqueous processing routes requires a robust scientific underpinning of all relevant processes. One key process is the clean-up of the contaminated salt from the electroreducing and electrorefining processes. A proposed method for this clean-up is zone refining, whereby the tendency of the contaminants to remain in the liquid phase during melting and freezing is exploited to ‘sweep’ the contaminants to one end of the sample. Experiments were performed, utilising off-the-shelf laboratory equipment, to demonstrate the feasibility of zone refining for clean-up of electroreducing and electrorefining wastes. This was successful for the electrorefining simulant samples, with effective segregation coefficient, *k_eff_*, values, which provide a measure of the degree of separation in the sample, between 0 and 1. Lower values indicate greater separation, with values of as low as 0.542 achieved here, corresponding to a reduction in RECl_3_ content from 10.0 wt.% to 8.4 wt.% (for 80% salt reuse). Due to difficulties in obtaining a fully homogeneous electroreducing simulant waste, it was not possible to demonstrate the feasibility of zone refining using the current experimental setup. Further research is required to elucidate the correct preparation conditions for production of homogeneous electroreducing waste simulants.

## 1. Introduction

Nuclear power has remained one of the most significant modes of power generation since its inception in the 1950s [1]. However, one of the key challenges in its continued use is waste management. Countries generally operate either an open or closed fuel cycle [2]. In an open fuel cycle, the nuclear fuel is used within the power plant and then is disposed of once it is spent. Conversely, in a closed fuel cycle, once the fuel is spent, it is removed from the power plant and reprocessed, with some or all of the fuel being reused to generate power [3]. Most countries which reprocess their spent nuclear fuel (SNF) utilise aqueous processes, e.g., PUREX (plutonium and uranium recovery by extraction), where solvent extraction combines the use of acids and organic complexants to dissolve the SNF and separate the actinides from the fission products [4]. However, there are some disadvantages to aqueous processes, such as their vulnerability to proliferation and radiation damage of the solvents [5].

Molten-salt-based electrochemical reprocessing (also known as pyroprocessing) solves these issues, as it is inherently proliferation-resistant and the inorganic electrolytes are not vulnerable to radiation damage [6]. The US developed an electrochemical process for the reprocessing of spent metallic fuel from sodium-cooled fast reactors, such as EBR-II [7]. Its application is being expanded to process spent fuels from oxide fuel reactors [6]. South Korea also utilises a molten-salt-based process, with the Korea Atomic Energy Institute (KAERI) beginning development of the process in 1997 [8]. Generally, the primary stage of pyroprocessing is electrorefining, where a metallic feedstock is dissolved in LiCl-KCl eutectic (LKE; 44:56 wt%) salt, which melts at 355 °C, and the re-useable actinides (mainly U and Pu) are deposited at multiple cathodes [4]. Due to the requirement of a metallic feedstock for electrorefining, an additional stage, known as electroreduction, is required for processing of oxide fuels [8]. In electroreduction, the oxide fuels are dissolved into a LiCl molten salt, with the oxygen transported to the anode and released [8]. In both processes, highly radioactive fission products build up in the salt as the useful actinides are electrochemically extracted, leaving the fission products to accumulate, requiring further treatment. These salts become rich in alkalis (Cs, Rb), alkaline earths (Ba, Sr), and rare earths (La, Ce, Pr…) [9]. To operate efficient and successful pyroprocessing of spent nuclear fuel, the LiCl and LKE molten salts used in electroreducing and electrorefining need to be periodically treated post-use to remove fission products and other impurities [4]. There are two primary reasons for this: (1) removal of the fission products means that a significant volume of the salts can be reused, increasing the efficiency of the system; and (2) chloride wastes tend to have low solubility in most common waste forms—reducing the volume of chloride waste through treatment will drastically reduce final waste volumes [4].

Recent research efforts, primarily out of South Korea and the USA, have worked to establish the applicability of zone refining, and other melt crystallisation techniques, for the treatment of pyroprocessing salt wastes [3,4]. The melt crystallisation techniques investigated include zone freezing [10], layer crystallisation (or coldfinger crystallisation) [10,11,12], the Czochralski method [13], and zone refining [6,13]. In general, this research has focused on the separation of CsCl and SrCl_2_ from LiCl, i.e., refinement of electroreducing waste salt where Cs-137 and Sr-90 will be the main fission product contaminants. This is due to the difficulty of separating CsCl and SrCl_2_ from LiCl using common techniques such as precipitation [10]. Cho et al. demonstrated separation efficiencies for Cs and Sr of >90% for both the zone freezing and layer crystallisation methods, provided the process rate (crucible rising velocity for zone freezing, crystal growth rate for layer crystallisation) was suitably slow (1.7 mm/h and <5 g/min, respectively) [10]. In general, the separation efficiency was similar for Cs and Sr. Cho et al. confirmed the efficacy of the layer crystallisation method, observing >90% separation efficiencies for Cs and Sr up to a crystal growth rate of 5 g/min, although the results suggest a greater efficiency of Cs separation compared to Sr separation [11]. Versey et al. also observed effective separation of Cs from LiCl using layer crystallisation, and they demonstrated that the efficiency can be maximised through increasing the cooling gas flow rate and crystal growth time [12]. The Czochralski method was investigated by Lee et al., with effective separation of Cs and Sr from LiCl observed, particularly at lower extraction speeds (2.5 mm/h) [13]. However, the calculated segregation coefficient (*k*) values suggest that Cs was separated more efficiently than Sr using this method. Shim et al. investigated zone refining for separation of Cs and Sr from both LiCl and LKE [14,15]. The efficiency of separation was inversely proportional to the freezing rate, and proportional to the number of passes. Cs was separated more effectively than Sr, but both elements were more difficult to separate from the LKE than from the LiCl salt. It was also found that the elements were more difficult to separate in LiCl-KCl-CsCl-SrCl_2_ compared to LiCl-KCl-CsCl or LiCl-KCl-SrCl_2_.

**Figure 1:**
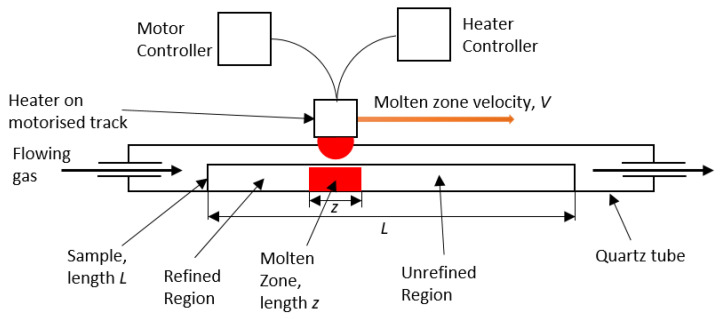
Schematic of zone-refinement apparatus.

One key issue for melt crystallisation techniques for processing of pyroprocessing wastes is their relatively slow rates. For a recovery yield of 60% LiCl from an LiCl-CsCl-SrCl_2_ waste, zone refining would take around 80 h, zone freezing would take around 102 h, and the Czochralski method around 52 h [14]. However, the advantage of the zone-refining technique is that multiple heating zones can be implemented, which increases the throughput by reducing the number of passes required to refine the waste salt [14]. Whilst there have been several investigations into melt crystallisation techniques for electroreducing waste salt [6,13], the same cannot be said for its electrorefining counterpart, where various rare-earth radionuclides will be the main impurities. This is likely due to the efficacy of other separation methods, such as reactive precipitation and reactive distillation [4]. However, the study of zone refinement for electrorefining waste treatment is of value for comparison to other methods. One proposed method of molten salt waste treatment is zone refining [8]. This technique was developed by W. G. Pfann at Bell Labs in the early 1950s [16], and can be used on any system with a non-zero segregation coefficient, *k*, as calculated by

(1) $$k=\frac{C_{S}}{C_{L}}$$

where *C_S_* is the concentration of the impurity/impurities in the solid salt, and *C_L_* is the concentration of the impurity/impurities in the molten salt. A schematic of this method can be seen in Figure 1. A moving heater generates a moving molten zone, travelling from one end of the sample to the other. When *k* < 1, as the salt behind the moving molten zone solidifies, the impurities remain within the molten zone at a higher concentration than in the recrystallised salt. Thus, as the molten zone travels along the sample it ‘sweeps’ the impurities to the end of the sample [14]. This method has most commonly been used in the preparation of high-purity materials for the production of semi-conductors [17,18,19,20] and scintillators [21,22].

The theoretical distribution of an impurity in a 1D zone-refining system can be calculated through several different equations based on the region within the sample. For a 1D system in equilibrium with sample length, *L*; position, *x*; normalised position *X = x/L*; molten zone length, *z*; and normalised molten zone length, *Z = z/L*, the sample can be split into three regions: 0 ≤ *X* ≤ 1 − *Z*, 1 − *Z* ≤ *X* < 1, and *X* = 1 [15]. For a single-pass system, the impurity distribution in the 0 ≤ *X* ≤ 1 − *Z* region can be calculated by:

(2) $$C_{S}\left(X\right)=C_{0}\left(1-\left(1-k\right)exp\left(-\frac{kX}{Z}\right)\right)$$

where *C_S_*(*X*) is the concentration of the impurity at position *X* and *C*_0_ is the initial impurity concentration [16]. In the 1 − *Z* ≤ *X* < 1 region, no new material is added to the molten zone and the impurity distribution is calculated by [15]

(3) $$C_{S}\left(X\right)=C_{0}\left(1-\left(1-k\right)exp\left(-\frac{k\left(1-Z\right)}{Z}\right)\right){\left(1-\frac{\left(X-\left(1-Z\right)\right)}{Z}\right)}^{k-1}$$

In the region *X* = 1, for *k* < 1, *C_S_*(*X*) approaches infinity; for *k* = 1, *C_S_*(*X*) = *C_0_*; and for *k* > 1, *C_S_*(*X*) = 0.

However, realistic systems are not in equilibrium, and so the segregation coefficient, k, must be replaced with an effective segregation coefficient, *k_eff_*. This can be calculated through the Burton–Prim–Slichter (BPS) theory [23,24]:

(4) keff=kk+1−kexp−VδD

where *V* is the velocity of the molten zone; *δ* is the diffusion layer thickness at the solidification interface; and *D* is the impurity diffusion coefficient in the liquid. Thus, the separation efficiency of zone refining is positively correlated with increasing impurity diffusion coefficient and decreasing molten zone velocity and diffusion layer thickness.

For a multi-pass, 1D, non-equilibrium system, the sample is broken up into four regions: *X* = 0, 0 < *X* < 1 – *Z*, 1 − *Z* ≤ *X* < 1, and *X* = 1. The impurity concentration at *X* = 0 after n passes of the heater, *C_S_^n^*(*X*), is given by

(5) $$C_{S}^{n}=k_{eff}\left(\frac{dx}{Z}\right)\left(\sum_{q=0}^{M_{1}-1}C_{S\left(q\;dx\right)}^{n-1}\right)$$

where *dx* is an individual element of length; *M_1_* is the control volume, equal in length to *Z*; and *q* is the index of the dx elements. In the 0 < *X* < 1 − *Z* region, the impurity distribution can be calculated by [25]

(6) $$C_{S\left(X\right)}^{n}=C_{S\left(X-dx\right)}^{n}+\left(\frac{kdx}{Z}\right)\left(C_{S\left(X+Z-dx\right)}^{n-1}-C_{S\left(X-dx\right)}^{n}\right)$$

In this region, the molten zone length is constant and re-solidification of the salt occurs on the left-hand side of the molten zone. In the last zone length section, 1 − *Z* ≤ *X* < 1, the heater begins to travel past the end of the sample and so no further material is added to the molten zone as re-solidification continues to occur on the left-hand side of the molten zone. In this region, the impurity distribution is given by [26]

(7) $$C_{S\left(X\right)}^{n}=\left(1+\left(\frac{1-k}{1-X}\right)dX\right)C_{S\left(X-dx\right)}^{n}$$

where *dX* is the displacement between each calculated data point. The impurity concentration where the molten zone fully passes the sample (*X* = 1) is calculated by [26]

(8) $$C_{S_{1}}^{n}=\frac{1}{dX}C_{0}-\sum_{X=0}^{X=1-dX}C_{S_{\left(X\right)}}^{n}$$

In this work, benchtop apparatus was used to conduct zone-refinement procedures using simulated electrorefiner and electroreducer wastes. These experiments were performed to demonstrate the feasibility of this zone-refining technique for the clean-up of electroreducing and electrorefining wastes. Comparing the experimental data with the theoretical behaviours described through Equations (1)–(8) will illustrate the effective segregation coefficient, *k_eff_*, as a measure for the degree of separation in the sample that was achieved. By demonstrating the suitability of laboratory-scale instrumentation, more progress can be made towards resolving the issues of appropriate waste management [27] for the nuclear sector, enabling more widespread use of nuclear power stations.

## 2. Materials and Methods

### 2.1. Materials and Sample Preparation

Raw materials of appropriate purity were obtained from Better Equipped, Staffordshire, UK (BE) and Alfa Aesar, Ward Hill, MA, USA (AA) (see Appendix A for details). All chemicals were kept in their original, sealed containers before use. Upon being opened, the containers were then stored within vacuum desiccators (with silica gel desiccant) to reduce hydration of the chlorides, which are generally hygroscopic. All weighing and mixing of chlorides took place within an Inert Corp, Amesbury, MA, USA, PureLab HE glovebox. To produce a salt sample for testing, the appropriate chemicals were placed inside the glovebox and the correct amounts were weighed out into an alumina BS111 crucible boat from Almath Crucibles Ltd., Newmarket, UK. The crucible boat was removed from the glovebox and placed inside the tube furnace (see Section 3.2) at a predetermined position. The furnace was heated at 2 °C/min to a temperature above the melting temperatures of the waste salt simulants, held for several hours (depending on sample), and then cooled back to room temperature at 2 °C/min, all while under a steady, low flow of N_2_.

Table 1 shows the three simulant salt waste compositions derived through consultation with NNL, based on their published example compositions [9]. Electroreducer waste is generated during the reduction of spent oxide fuels (UO_2_, MO_x_) to produce a metallic feed for electrorefining. The ‘No-Cs’ and ‘Full-Cs’ variants are based on whether a high-temperature decladding process is or is not used, respectively, which may result in the volatilisation of Cs [28]. The electrorefiner waste is an estimate of the salt composition after the residual actinides have been removed. Zone-refining experiments were performed on the electrorefiner waste simulant and the Full-Cs electroreducer waste simulant.

### 2.2. Experimental Setup

**Figure 2:**
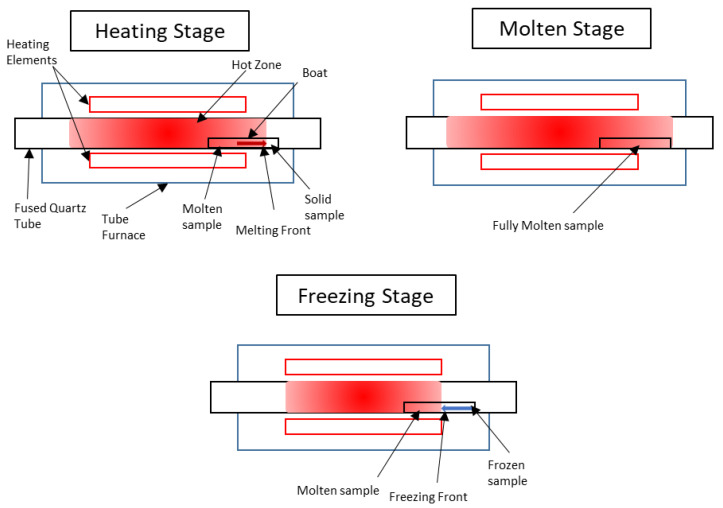
Schematics showing normal-freezing process of zone refining.

Zone-refining experiments were performed in an Elite Furnaces, Market Harborough, UK, 12/100/750 tube furnace with a fused quartz tube and flowing nitrogen. Due to the layout of the heating elements within the furnace (one controllable heating zone in the centre of the furnace), zone-refining experiments were performed in accordance with the schematics shown in Figure 2. The sample boat is placed off-centre in the furnace and during the heating stage, the end of the sample closest to the centre begins to melt, and this melting front continues across the sample until it is entirely molten (molten stage). Once the sample is fully molten, the temperature of the furnace is decreased at a controlled rate, such that a freezing front passes from the end of the sample furthest from the centre of the furnace to the end of the sample closest to the centre of the furnace (freezing stage). This is equivalent to zone refining with a molten zone which covers the entire sample, i.e., *Z* = 1. The theoretical impurity distributions for *Z* = 1 experiments are discussed in Section 2.4.

**Figure 3:**
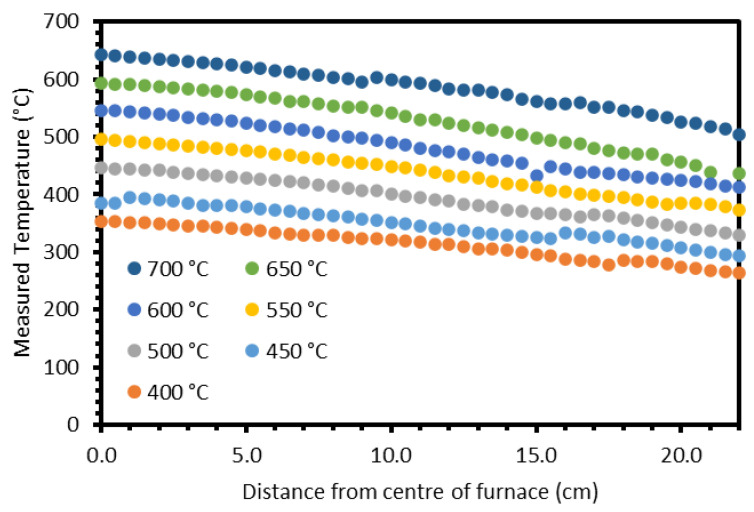
Measured temperature profiles within the Elite tube furnace as a function of set temperature and position.

Prior to beginning zone-refining experiments, the temperature profile of the furnace as a function of the controller set temperature and position must be measured. To achieve this, the temperature of the furnace was set to a temperature of 400 °C–700 °C in 50 °C increments, at a heating rate of 2 °C min^−1^ and allowed to equilibrate for 30 min. The temperature from the centre point to the end of the tube was then measured in 0.5 cm increments using a steel thermocouple (TC Direct, Uxbridge, UK, 310 Stainless Steel) and handheld reader. The measured temperature profiles are shown in Figure 3, and temperatures measured at 0.0 cm are reported in Appendix A, Table A3. The general trend in the profiles is for the measured temperature to decrease as the thermocouple is moved further from the centre of the furnace, as expected. There are some shifts in the data, e.g., at 9.5 cm in the 700 °C profile, which are likely due to a slight change in the position of the thermocouple within the cross-section of the tube. To obtain more consistent data, these shifts were corrected by a constant factor, i.e., where a profile increased instead of decreased, every data point after that was offset by the same value. The profiles were then fit with a second-order polynomial fit of the form $ax^{2}+bx+c$. The parameters for each profile are provided in Table 2. The positions of 10 °C isotherms (640 °C, 630 °C, 620 °C, etc.) for each profile were noted, and the change in position of these isotherms between profiles was calculated. These isotherm position changes were then converted to rates of isotherm position change as a function of the change in furnace set temperature. These rates can then be used to estimate freezing-front velocities through Equation (9):

(9) $$V=r_{I}\times r_{T}$$

where *r_I_* is the rate of isotherm position change in cm/°C; and *r_T_* is the set cooling rate of the furnace in °C/s. The minimum value of *r_T_* for the furnace is 0.0017 °C/s (0.1 °C/min) and the highest recommended value is 0.0333 °C/s (2.0 °C/min) in order to avoid thermal shock of the alumina sample boats. The calculated average rates of isotherm position change vary between 0.103 cm/°C and 0.168 cm/°C. The estimated range of possible *V* values is 0.00018 cm/s–0.00559 cm/s (0.108–3.354 mm/min), which compares well with the values used previously by Shim et al. (0.25–3 mm/min) [14,15].

For the zone-refining experiments, the samples (produced according to Section 2.1) were placed in an alumina boat, and then, into the tube, with the axial position of the boat along the furnace being recorded. The end caps were then fixed to the fused quartz tube, and the system was purged with N_2_ for 30 min. A low flow of N_2_ was continued for the rest of each experiment. Once the system was purged, the set temperature of the furnace was increased at 2 °C min^−1^ up to the target temperature. Once the set temperature had been reached, it was held for a period of time to allow the temperature to equilibrate. The set temperature was then reduced at a constant rate back to room temperature, such that the sample froze from one end to the other. From the rate of temperature decrease and the measured temperature profiles at different set temperatures, an estimate of the velocity of the freezing front was obtained. Once the furnace reached room temperature, the sample was removed and stored inside a desiccator prior to preparation for analysis.

### 2.3. Initial Testing

Prior to performing zone refining on samples containing the electrorefining simulant, initial tests were performed to assess the experimental setup. A number of these initial tests were carried out, using LKE with Ba or Cs as a tracer of refinement. Zone refining was performed as described in Section 2.2. The details for each test are shown in Table A2. Once each sample had undergone the zone refining, 1–2 cm sections were taken from each end of the sample using a Top Tech Preciso low-speed saw with diamond-coated abrasive cutting wheel. These sections were then sectioned longitudinally using the saw. The longitudinal sections were mounted in Buehler EpoThin resin, ground to a P1200 SiC paper finish, and then carbon-coated and painted with Silver DAG to ensure sample conductivity. The resin-mounted samples were then analysed by SEM-EDX (FEI, Hillsboro, OR, USA, Quanta 650 SEM and Oxford Instruments, Abingdon, UK, X-Max 80 EDX detector) to determine sample homogeneity and ascertain the degree of refinement achieved.

### 2.4. Full Simulant Zone-Refining Experiments

#### 2.4.1. Electrorefining Simulant

Full electrorefining simulant samples were prepared as described in Section 2.1, and zone refining was performed in accordance with Section 2.2. For zone refining, each sample was placed with its inner end 5.0 cm from the centre of the furnace. Table 3 shows the target temperature for each sample, the estimated temperatures at the centre and ends of the boat, and the rate of temperature decrease and estimated freezing-front velocity for each sample. Samples were held under vacuum in desiccators upon removal from the tube furnace. Note that the nomenclature “FS” in the sample names refers to the compositions being those of their respective full waste simulants, as given in Table 1, where “LKE-” denotes electrorefining samples and “L-” denotes electroreducing samples.

After removal from the desiccator, the samples were cross-sectioned every 0.5 cm along their length using the low-speed saw. Each section of sample was weighed, and then, dissolved in 5 mL of 1% HNO_3_ solution made with UHQ Type II water. The dissolved samples were sent to the MIDAS facility at the University of Sheffield for ICP-OES analysis to determine the concentrations of Li, K, Y, La, Ce, Pr, Nd, and Sm as a function of position along the sample. The mg/L concentration data received were normalised to the mass of each section and converted to an equivalent mg per kg of sample value. One section of each sample was retained for SEM-EDX analysis to assess sample homogeneity.

#### 2.4.2. Electroreducing Simulant

The two electroreducer samples were prepared in the same manner as the electrorefining samples. The samples were melted for 5 h at 620 °C before undergoing zone refining (see parameters in Table 4). As with the electrorefining samples, the samples were positioned 5.0 cm from the centre of the tube furnace, held under vacuum upon removal from the furnace, and analysed using ICP-OES (for Li, K, Cs, Rb, Ba, and Sr) and SEM-EDX.

### 2.5. Impurity Distribution Modelling

As noted in Section 2.2, due to the arrangement of the heating elements within the furnace, the zone-refining experiments were performed with a normalised molten zone length, *Z*, of 1. This means that the entire molten sample was subject to normal freezing and can therefore be modelled using the normal-freezing equations, as defined by Shim [14,15]. The impurity distribution for a region of normal freezing is given by Equation (3). Substituting *Z* = 1 into Equation (3) and using *k_eff_* for a non-equilibrium system gives

(10) $$C_{S}\left(X\right)=C_{0}k_{eff}{\left(1-X\right)}^{k_{eff}-1}$$

Thus, the impurity distribution is a function of *X*, which is independent of the experimental conditions; *C*_0_, which is user-controlled and defined by the batching of the salt mixture; and *k_eff_*. In turn, *k_eff_* is a function of *k*, which is dependent on the salt system; *V*, which is user-controlled through the furnace setup; *δ*, which can be reduced through improved mixing; and *D*, which is a fundamental property of each element that is being separated from the base salt (LiCl or LKE). The quantitative effect of each of these parameters can be demonstrated by varying each one in turn and plotting the values returned by Equation (10). Figure A9, Figure A10, Figure A11 and Figure A12 in Appendix A show the effects of *k*, *V*, *δ*, and *D*, respectively, on the impurity distribution profile of a theoretical 1D system.

Figure A9, Figure A10, Figure A11 and Figure A12 show that *C_S_*(*X*) is most sensitive to variation in *k*, with a higher value of *k* increasing segregation, as expected, and less dependent on factors pertaining to a particular salt system or element being separated. This should enable the estimation of *k* by comparison of experimental and modelled data. The next most influential parameter on *C_S_*(*X*) is *V*, where a lower value of *V* is preferable for greater segregation of impurities; however, there must be a compromise between the efficiency of the process and the duration of the process. The sensitivity of *C_S_*(*X*) to *δ* is lower than for *k* and *V*, and as a measure of the degree of mixing within the molten zone it is likely that it will remain constant between different tests within the same experimental framework. The sensitivity of C_S_(X) to the diffusion coefficient of the impurity was assessed through values for five different rare-earth chlorides (LaCl_3_, CeCl_3_, NdCl_3_, DyCl_3_, and SmCl_3_) in LKE taken from the literature [6]. Although the values of *D* span 1–2 orders of magnitude, there is little change in C_S_(X), with the relative standard deviation between the five profiles being between 1 and 2%. This suggests that the efficiency of the segregation can be maintained despite varying salt waste compositions.

## 3. Results

### 3.1. Initial Testing

**Figure 4:**
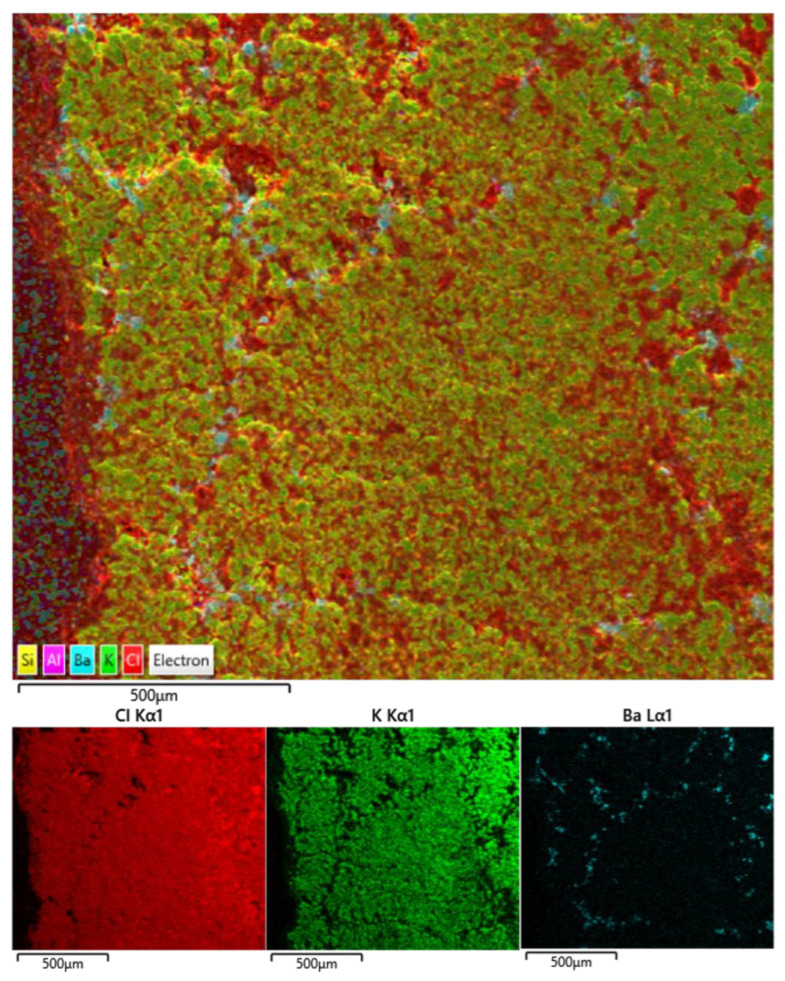
Cl, K, and Ba EDX maps of LKE-Ba5 (100× magnification).

**Figure 5:**
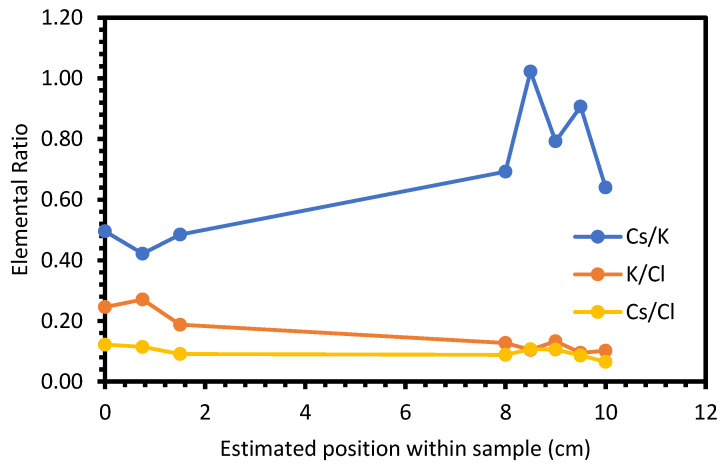
Cs/K, K/Cl, and Cs/Cl ratios of the LKE-Cs5_b sample, as measured by SEM-EDX, as a function of position along the sample.

The first four test samples utilised BaCl_2_ as the tracer for refinement. However, there appeared to be issues with the BaCl_2_ dissolving within the LKE. This was confirmed by SEM-EDX (Figure 4), which showed small, highly concentrated regions of BaCl_2_ within a matrix of KCl (N.B., Li is not detectable through EDX due to a low Z number). CsCl was therefore used as the tracer of refinement for the last two initial tests. SEM-EDX on the final sample showed some evidence of refinement of the LKE, with the Cs concentration significantly greater closer to the centre of the furnace (Figure 5). The data are taken from spectra collected for 10 min at ×150 magnification from the whole area of the image. Based on this result, it was deemed reasonable to move forward to zone-refining experiments on full simulant samples.

### 3.2. Electrorefining Simulant Zone Refining

**Figure 6:**
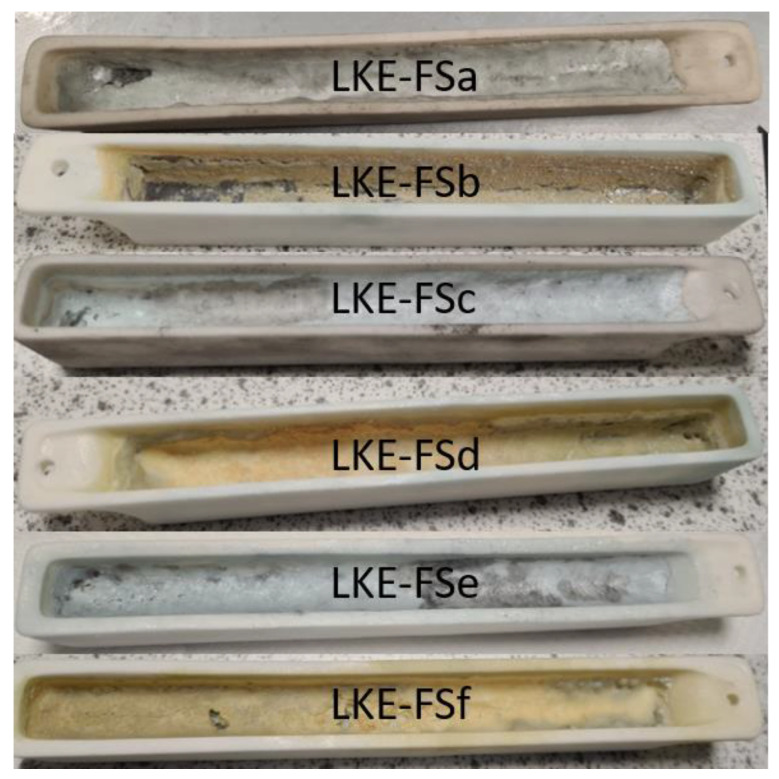
Images of electrorefining simulant samples following zone refinement.

**Figure 7:**
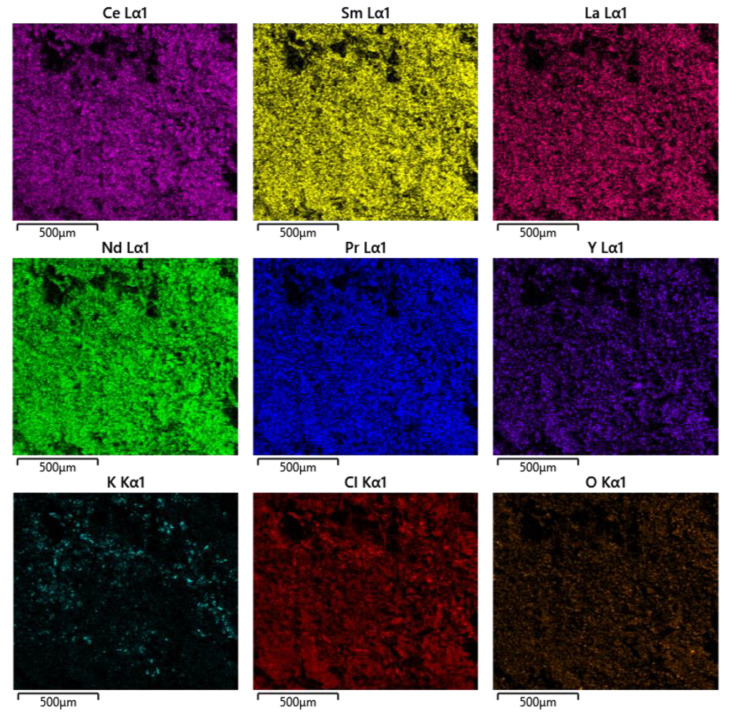
EDX maps of unvolatilised residue from LKE-FSb zone-refined sample.

Of the six full electrorefining simulant samples, two suffered from significant volatilisation (LKE-FSb and LKE-FSd, as shown in Figure 6). These samples underwent zone refining with the slowest cooling rates, and therefore, remained at higher temperatures for longer periods of time, which is the likely cause of the increased volatilisation. An SEM-EDX analysis of the residue remaining on the alumina boat from the LKE-FSb sample showed that it consisted of rare-earth (RE) chlorides, with little K present (Figure 7). In addition, O is observed in the data, which might suggest the formation of RE oxychlorides. This could explain why this residue remains; RE oxychlorides are prone to form in the presence of moist air [29], which would occur to a greater extent when cooling over longer periods.

**Figure 8:**
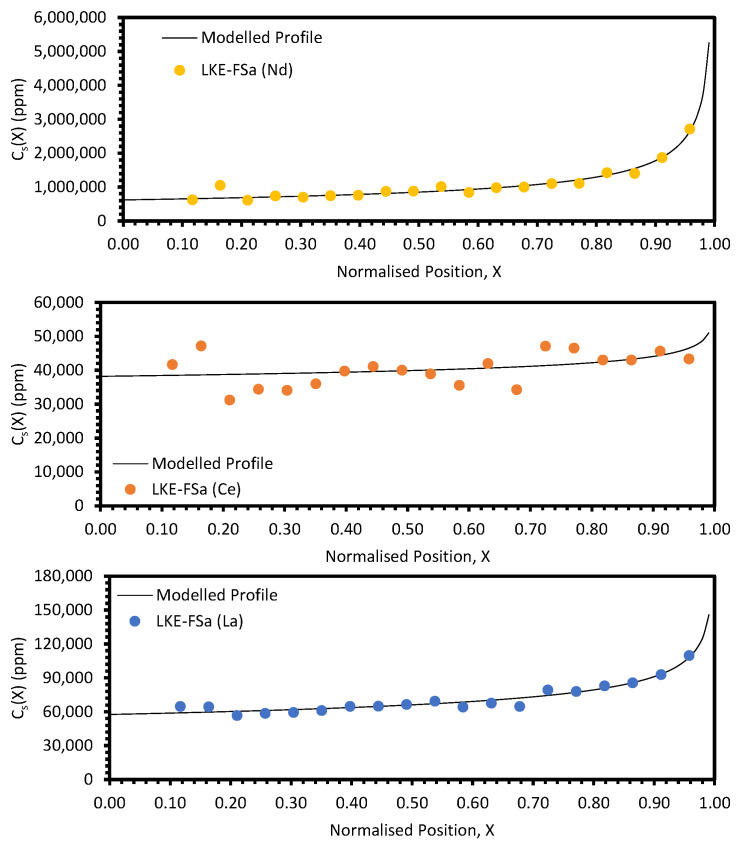
Experimental distribution profiles for Nd, Ce, and La for the LKE-FSa sample compared to modelled profiles.

**Figure 9:**
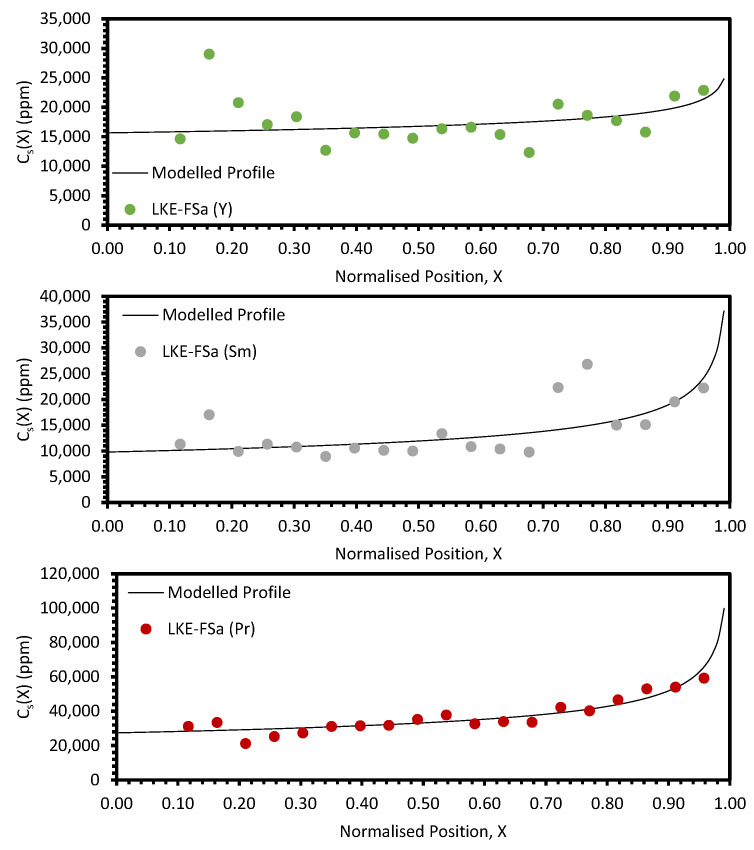
Experimental distribution profiles for Y, Sm, and Pr for the LKE-FSa sample compared to modelled profiles.

The post-refinement LKE-FSa sample appeared to have a consistent eutectic composition across the full length, as shown by the average K/Li ratio measured by ICP-OES across the sample (0.46 ± 0.01). This suggests that the batched sample was well melted and mixed during the melting and zone-refining runs. The experimentally determined distribution profiles for the REs are shown in Figure 8 and Figure 9. Each experimental profile is fitted with Equation (10) (with *k_eff_* provided by Equation (4)). To achieve this, the squared residual, *R*^2^*,* for each data point was calculated by

(11) $$R^{2}={\left(\frac{E_{X}-C_{X}}{C_{X}}\right)}^{2}$$

where *E_X_* is the experimentally determined concentration at normalised position *X*; and *C_X_* is the calculated concentration at *X*. The Solver function in Microsoft Excel was then utilised to fit Equation (10) to the data, with a focus on minimising the average *R*^2^ value. *C_0_* and *k* were allowed to refine freely, whilst *V*, *D*, and *δ* were kept constant. *V* was calculated from the temperature gradient and cooling rate (see Section 2.2); the *D* values for Ce, Nd, Sm, and La were taken from Sridharan et al. [6], whilst those for Y and Pr were estimated to be of the same order of magnitude; and *δ* was chosen to be 0.001 cm as this represents a well-mixed molten phase, as would be expected from a sample which is fully molten before refinement [14].

The average *R*^2^ values for all samples can be found in Table A4 and the fitting parameters for the REs are shown in Table A5. This provides an estimate of *k_eff_* for each RE. The separation behaviour of the REs varied significantly, with Nd exhibiting the greatest separation (*k_eff_* = 0.542). Pr, Sm, and La all had *k_eff_* values between 0.714 and 0.801, but Ce and Y showed only minor separation, with *k_eff_* values greater than 0.900.

**Figure A1:**
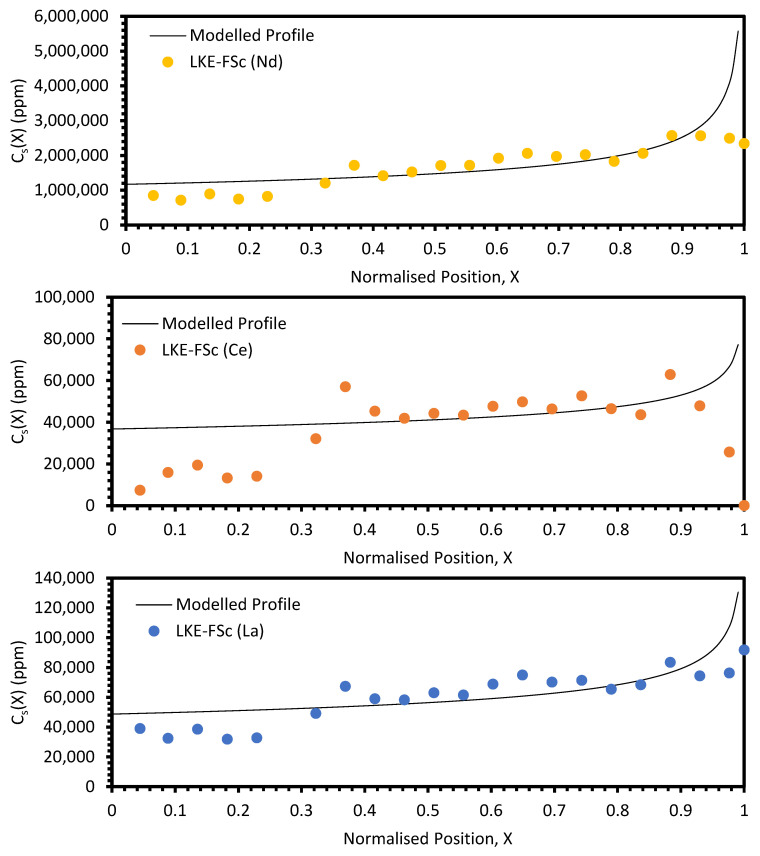
Experimental distribution profiles for Nd, Ce, and La for the LKE-FSc sample.

The LKE-FSc sample also showed a consistent K/Li ratio across the sample (0.50 ± 0.01), again suggesting that it was reasonably homogeneous. As with the LKE-Fsa sample, Nd showed the greatest separation (Figure A1). The behaviour of the other elements was more similar, with their *k_eff_* values falling between 0.737 and 0.852 (Table A6).

**Figure A3:**
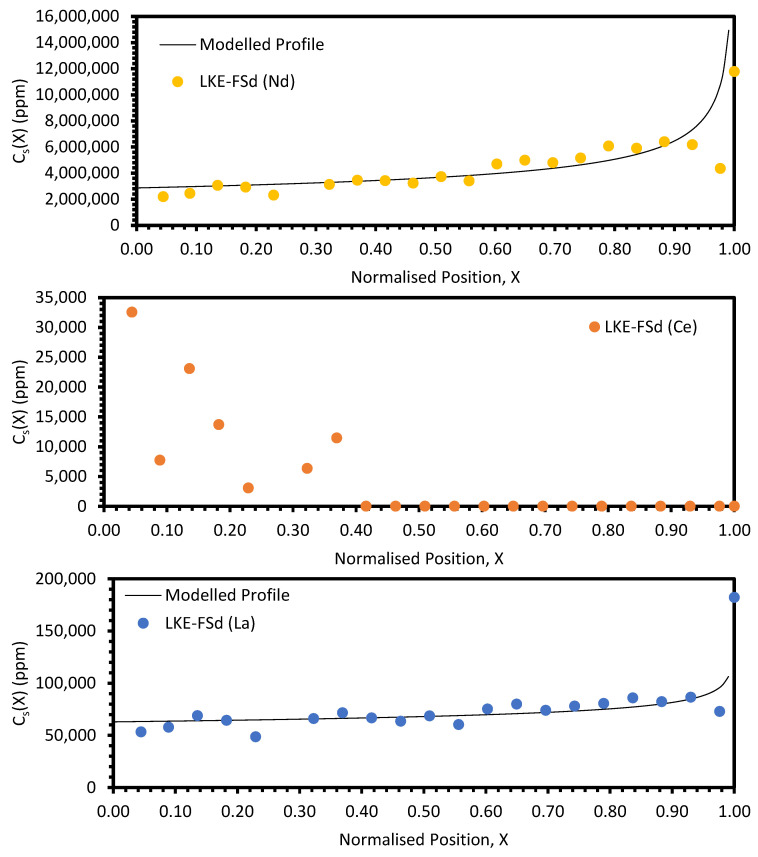
Experimental distribution profiles for Nd, Ce, and La for the LKE-FSd sample compared to modelled profiles.

**Figure A4:**
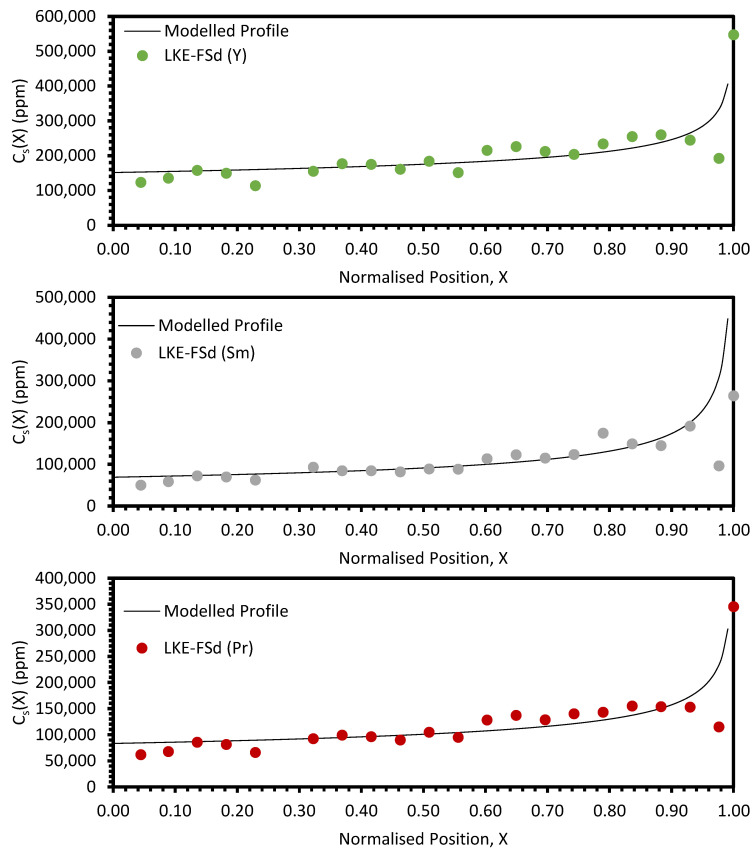
Experimental distribution profiles for Y, Sm, and Pr for the LKE-FSd sample compared to modelled profiles.

The average K/Li ratio for LKE-FSd was 0.56 ± 0.02, suggesting reasonable homogeneity. The data for Nd, Ce, and La are shown in Figure A3, and those for Y, Sm, and Pr are shown in Figure A4. Due to the volatilisation of the sample during zone refining, the mass of sample available per section for ICP-OES was limited. This resulted in many of the Ce data being below the limit of detection for the instrument, and thus, no information can be drawn from the remaining data. The model parameters are shown in Table A8. Nd and Sm had the lowest *k_eff_* values at 0.647 and 0.600, respectively. Pr and Y had similar *k_eff_* values (0.724 and 0.789), whilst La had a significantly higher value than the other elements (0.888).

**Figure A5:**
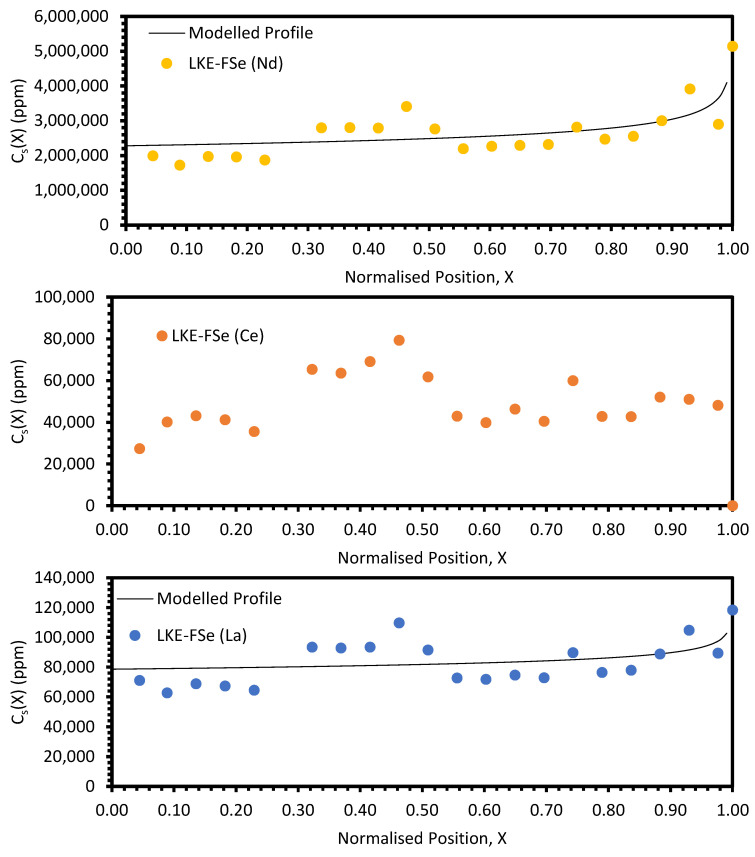
Experimental distribution profiles for Nd, Ce, and La for the LKE-FSe sample compared to modelled profiles.

**Figure A6:**
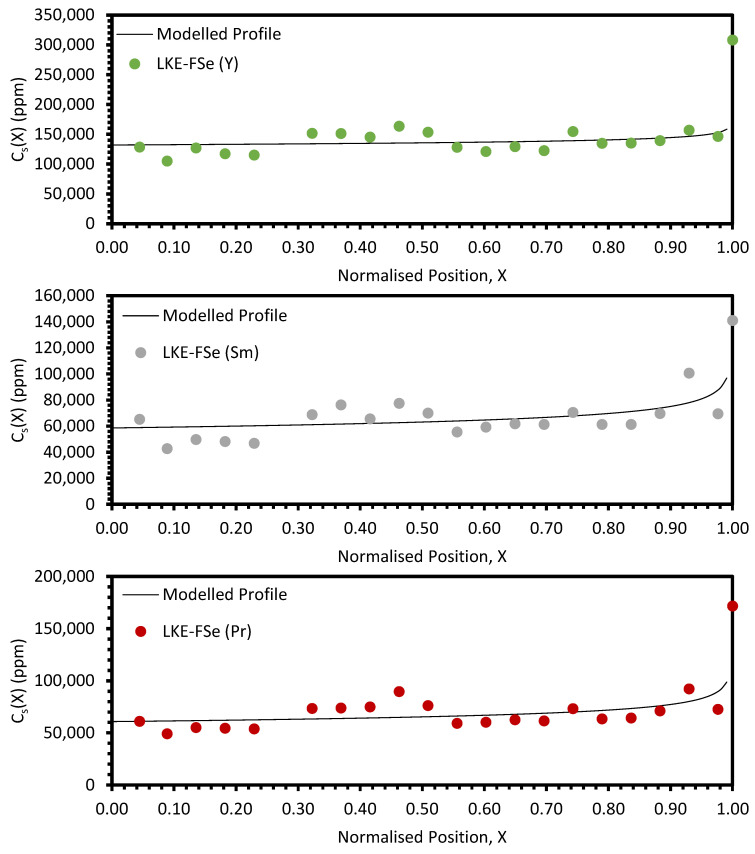
Experimental distribution profiles for Y, Sm, and Pr for the LKE-FSe sample compared to modelled profiles.

The LKE-FSe sample had an average K/Li ratio of 0.51 ± 0.03, suggesting reasonable consistency of composition across the sample. The experimental data and modelled profiles are shown in Figure A5 and Figure A6, with model parameters displayed in Table A9. Nd, Sm, and Pr exhibited very similar profiles, with calculated *k_eff_* values between 0.875 and 0.896. La and Y demonstrated flatter profiles with a lower degree of separation; the *k_eff_* values were 0.943 and 0.961, respectively. It was not possible to obtain a fit of the Ce data, suggesting that little to no refinement took place.

**Figure A7:**
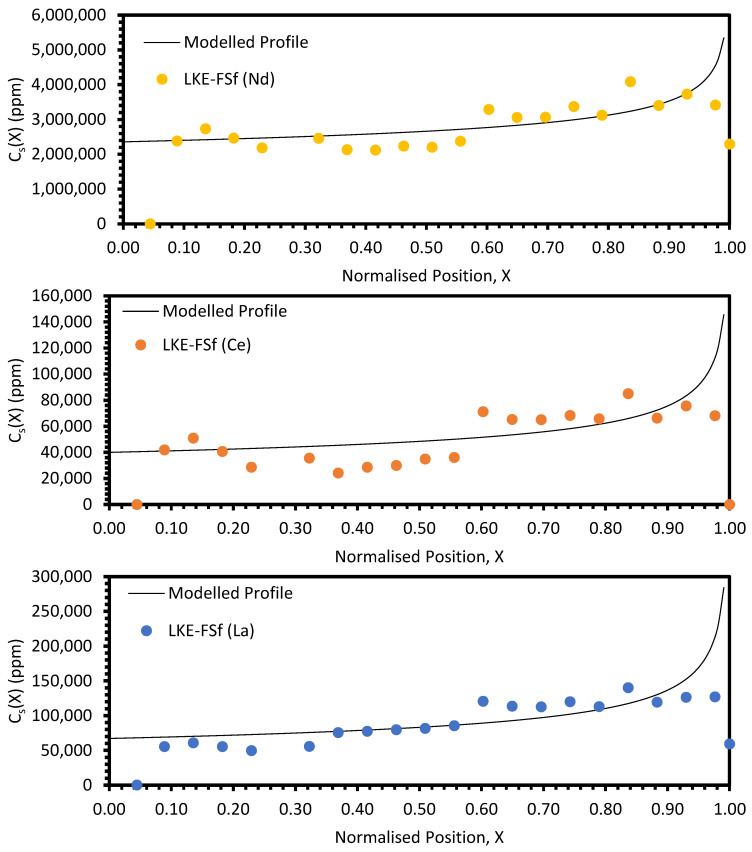
Experimental distribution profiles for Nd, Ce, and La for the LKE-FSf sample compared to modelled profiles.

**Figure A8:**
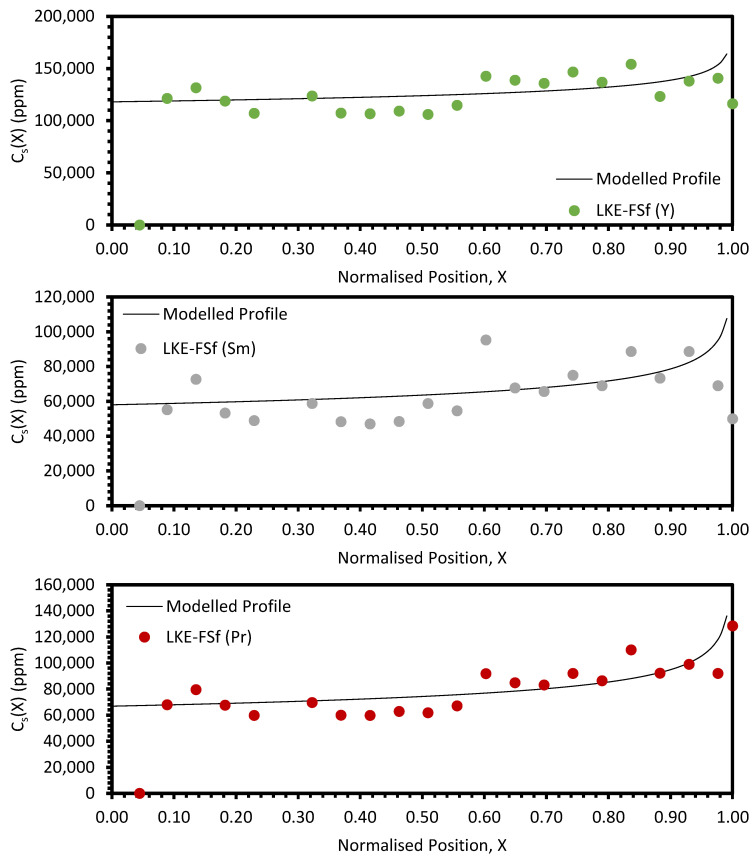
Experimental distribution profiles for Y, Sm, and Pr for the LKE-FSf sample compared to modelled profiles.

The LKE-FSf sample had an average K/Li ratio of 0.49 ± 0.04, suggesting reasonable consistency of composition across the sample, although the variation in this value with position was greater than for the other samples. The experimental data and modelled profiles for the REs are shown in Figure A7 and Figure A8, with the parameters for the modelled profiles shown in Table A10. La and Ce had the lowest *k_eff_* values, at 0.691 and 0.723, respectively. Nd, Sm, and Pr exhibited similar separation behaviour, with *k_eff_* values between 0.825 and 0.868, but Y showed a much lower separation level, with a *k_eff_* of 0.930.

### 3.3. Electroreducing Simulant Zone Refining

**Figure 10:**
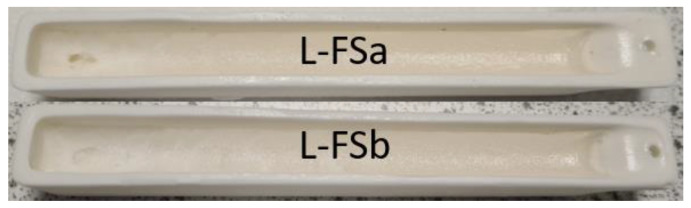
Images of electroreducing simulant samples following zone refinement.

The appearance of both of the electroreducing samples is consistent post-refinement, shown in Figure 10, with volatilisation at lower levels than for some of the electrorefining samples. The same elemental concentration profile fitting method was applied to the samples as was used for the electrorefining samples. The residual data for each fit can be found in Table A5 in Appendix A.

**Figure 11:**
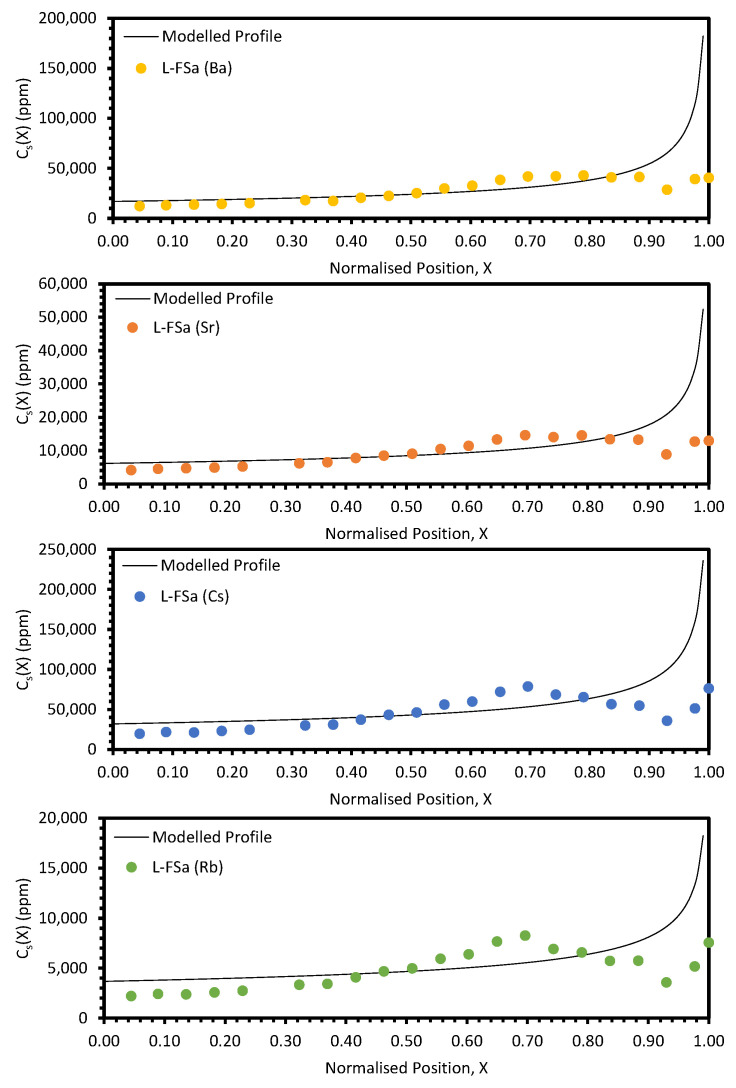
Experimental distribution profiles for Ba, Sr, Cs, and Rb for the L-FSa sample compared to modelled profiles.

**Figure 12:**
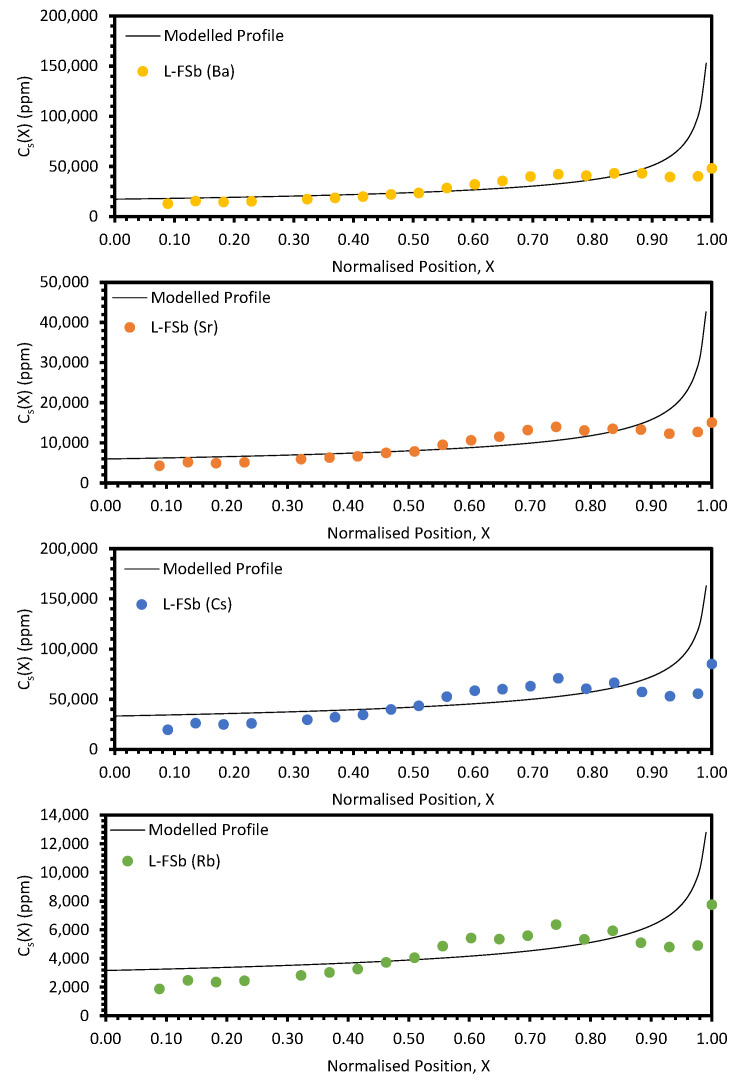
Experimental distribution profiles for Ba, Sr, Cs, and Rb for the L-FSb sample compared to modelled profiles.

The experimental data and modelled profiles for the L-FSa and L-FSb samples are shown in Figure 11 and Figure 12, respectively. The residuals for each fit were significantly higher for these samples compared to those for the electrorefining samples (see Table A4 and Table A5). There are also systematic deviations of the modelled profiles from the experimental data. In particular, for the regions *X* ≤ 0.40 and *X* > 0.80, the calculated values are consistently greater than the experimental values, whilst for 0.55 < *X* < 0.80 the calculated values are consistently underestimates when compared to the experimental values. Due to these factors, the *k_eff_* values generated by fitting with Equation (10) are suspect and cannot reasonably be used for comparison with the calculation of *DF_i_* values for refined and waste salt compositions.

## 4. Discussion

The approach taken in this work regarding the *k_eff_* values generated by fitting with Equation (10) was found to be unsuitable when considering the electroreducing wastes, as shown in Figure 11 and Figure 12. As such, further discussions will focus on the electrorefining simulant zone-refining efforts.

In the electrorefining simulant zone-refining data, the elemental concentration profiles for the RE elements show that some degree of zone refinement was achieved in all five analysed samples (due to volatilisation, ICP-OES analysis of the LKE-FSb sample was not possible). Using the calculated elemental concentration profiles for each element in each sample, decontamination factors and waste compositions can be calculated. The decontamination factor, *DF_i_*, for a given element, *i*, can be calculated by

(12) $${DF}_{i}=\frac{average\;concentration\;of\;i\;for\;X_{R}\leq X<1}{average\;concentration\;of\;i\;for\;0\leq X\leq X_{R}}$$

where *X_R_* is a predetermined ‘refinement boundary’. For 0 ≤ *X* < *X_R_*, the base salt has been refined to remove some of the impurities, and for *X_R_* ≤ *X* < 1, the impurities have been concentrated to produce a waste material. The average concentrations are calculated by evaluating the definite integral across the two intervals (0 ≤ *X* < *X_R_* and *X_R_* ≤ *X* < 1) and dividing by the interval width:

(13) $$average\;concentration\;of\;i=\frac{\int_{0}^{X_{R}}C_{S}\left(X\right)}{X_{R}}\;or\;\frac{\int_{X_{R}}^{0.99999}C_{S}\left(X\right)}{0.99999-X_{R}}$$

*X* = 0.99999 is taken to be a reasonable estimate of *X* = 1, as there is a singularity in C_S_(X) at *X* = 1. *DF_i_* values for *X_R_* = 0.8 and 0.9 are shown in Table 5 and Table 6, respectively. In general, the minimum enrichment of an RE in the ‘waste’ portion of the sample for *X_R_* = 0.8 is 8% (Y, LKE-FSe) and the maximum is 286% (Nd, LKE-FSa). The equivalent values for *X_R_* = 0.9 are 11% (Y, LKE-FSe) and 360% (Nd, LKE-FSa).

Based on the average elemental concentrations calculated through Equation (13), the composition of the refined salt and the waste salt can be estimated. Li, K, and Cl are assumed to remain in the same proportions throughout each sample. The estimated compositions of the refined and waste salts for *X_R_* = 0.8 and 0.9 are shown in Table 7, Table 8, Table 9 and Table 10. Due to the variation in *DF_i_* between the REs, their relative proportions vary between the samples, but an estimate of the overall compositional change can be made using the total wt.% of RECl_3_. The total RECl_3_ content of the waste salt was between 11.4 and 16.2 wt.% for *X_R_* = 0.8, and between 12.1 and 20.3 wt.% for *X_R_* = 0.9. These contaminant-enriched waste salts could be sent for immobilisation, or for further processing, e.g., phosphate precipitation of the RE ions from the chloride salt.

**Figure 13:**
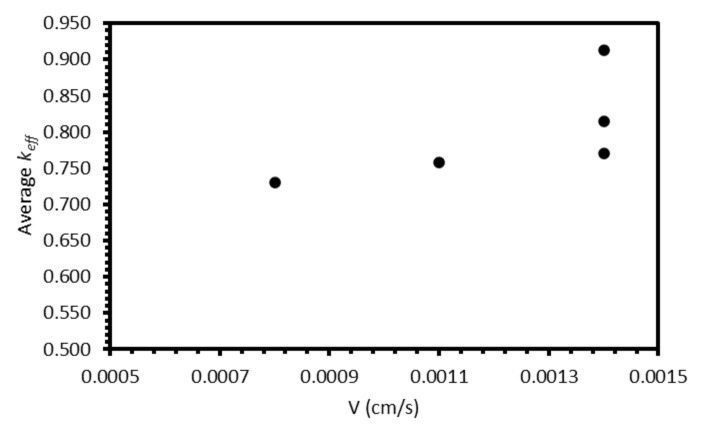
Average k_eff_ for each electrorefining sample vs. estimated freezing-front velocity, V.

**Figure A9:**
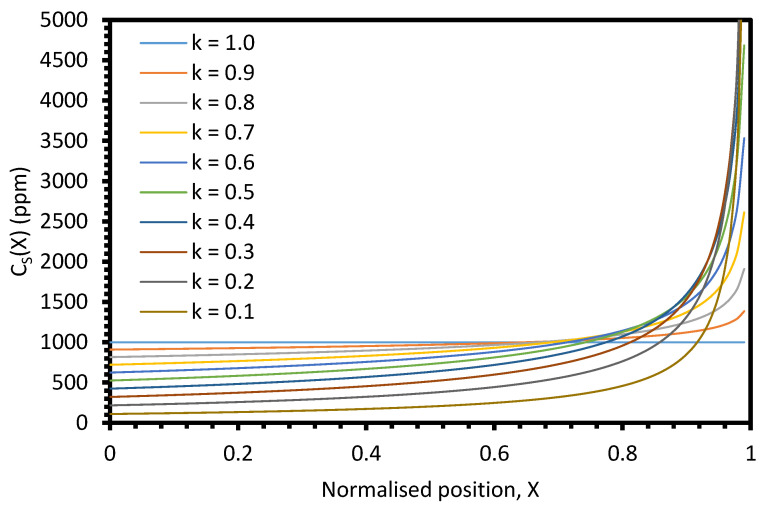
Dependence of C_S_(X) on k in a single-pass, normal-freezing experiment. Parameters used: L = 100 cm, Z = 1, C_0_ = 1000 ppm, V = 0.0001 cm/s, δ = 0.01 cm, D = 1.01 × 10^−5^ cm^2^/s.

**Figure A10:**
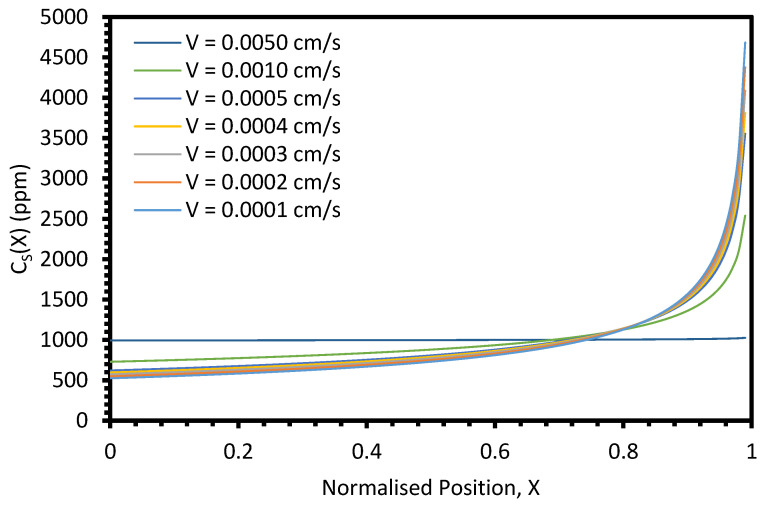
Dependence of C_S_(X) on V in a single-pass, normal-freezing experiment. Parameters used: k = 0.5, L = 100 cm, Z = 1, C_0_ = 1000 ppm, δ = 0.01 cm, D = 1.01 × 10^−5^ cm^2^/s.

**Figure A11:**
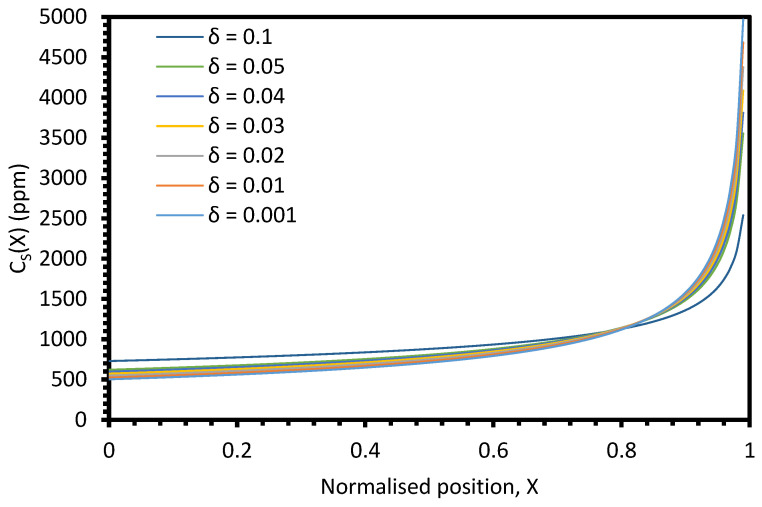
Dependence of C_S_(X) on δ in a single-pass, normal-freezing experiment. Parameters used: k = 0.5, L = 100 cm, Z = 1, C_0_ = 1000 ppm, V = 0.0001 cm/s, D = 1.01 × 10^−5^ cm^2^/s.

**Figure A12:**
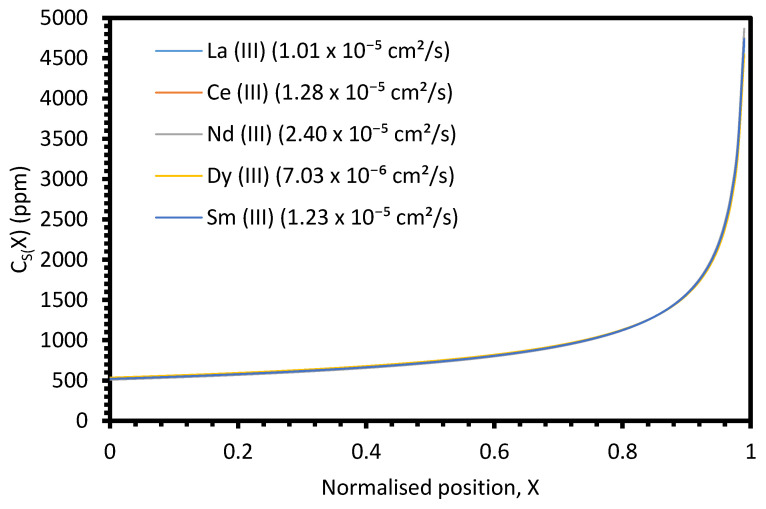
Dependence of C_S_(X) on D in a single-pass, normal-freezing experiment. The D values are those noted by Sridharan et al. for rare-earth chlorides [6]. Parameters used: k = 0.5, L = 100 cm, Z = 1, C_0_ = 1000 ppm, V = 0.0001 cm/s, δ = 0.01 cm.

The primary parameter varied between the six electrorefining simulant tests was the cooling rate of the furnace, and hence, the velocity of the freezing front, *V*. Based on Equation (4), we would expect *k_eff_* to decrease with decreasing *V*, assuming *k*, *δ*, and *D* remain constant. For the example system described in Figure A9 (*k* = 0.5, *L* = 100 cm, *Z* = 1, *C_0_* = 1000 ppm, *δ* = 0.01 cm, *D* = 1.01 × 10^−5^ cm^2^/s), the *k_eff_* value would be expected to decrease from 0.800 to 0.688 as *V* decreases from 0.0014 to 0.0008 cm/s (the velocity range of the successful experiments). However, when the average *k_eff_* values for the six elements for each test are plotted against the estimated *V*, the relationship is unclear (Figure 13). Whilst there may generally be a slight decrease in *k_eff_* with *V*, the range of the three *k_eff_* values at 0.0014 cm/s (0.144) is greater than the difference between *k_eff_* at 0.0008 cm/s (0.730) and the average of the three values at 0.0014 cm/s (0.833). Further experiments at a range of *V* values, with duplicate or triplicate data, are required to determine whether the relationship is present.

**Figure 14:**
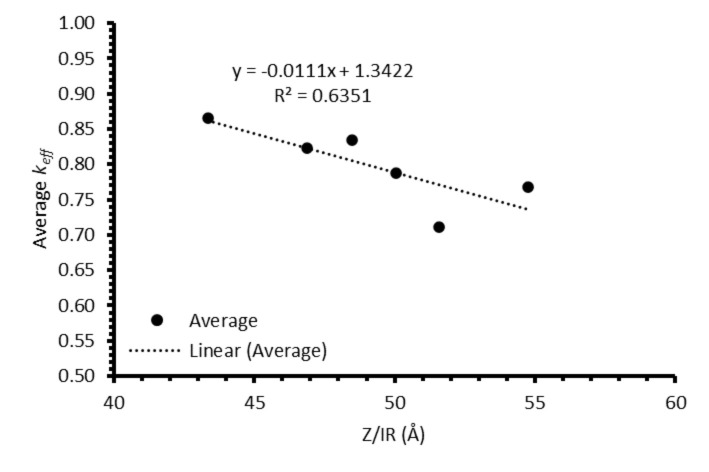
Average k_eff_ for each element against atomic number/ionic radius (Z/IR).

Local maxima in the distribution profiles around the normalised position 0.6 to 0.8, presented in Figure A7 and Figure A8, are indicative of incomplete refinement due to the single-pass experimental setup used. Further zone-refinement processing and the use of multi-pass modes could improve the final distribution profiles and result in profiles more consistent with the modelled profiles. However, the variation in the refinement behaviour of the REs is of interest as this will affect the efficacy of zone refining and the composition of the waste generated. The REs are chemically similar, with all six elements present in the salt as 3+ ions. However, there is a slight difference in their coordination within their respective chlorides, with Nd, Ce, La, Sm, and Pr nine-coordinated, whilst Y is six-coordinated. Plotting the average *k_eff_* for each ion against their atomic number divided by ionic radius (*Z*/*IR*) appears to provide a general linear trend of increasing separation with increasing *Z*/*IR*, although this should be caveated with an *R*^2^ value of 0.64 (Figure 14). Whether there is a physical explanation for such a relationship remains unclear and would require further work to elucidate.

## 5. Conclusions

Zone refinement of electrorefining simulant salt was partially achieved using an experimental setup consisting of off-the-shelf equipment. As far as the authors are aware, data on successful zone refining of electrorefining simulant salts using such equipment have not been published previously in the literature. The zone-refining experiments on electroreducing simulant salt samples were not successful, likely due to the contaminant salts (particularly BaCl_2_ and SrCl_2_) not dissolving into the base LiCl salt. This work sets out a framework for future work, whereby experimental data can be assessed through the appropriate theoretical models. Further work is required to identify the optimum processing parameters for the electroreducing simulant salt. The experiments were performed in a ‘normal freezing’ arrangement, which allowed for single-pass experiments only. It is anticipated that by improving on the methods herein, and expanding the methods into multi-pass models, as described by Spim [25], molten salt zone refinement research can be accelerated significantly.

Clean-up of salt wastes is a key pillar for successful implementation of pyroprocessing for used nuclear fuel. It determines salt reuse rates and waste volumes and compositions, and thus, the work in this report is a vital part of understanding a potential process within salt clean-up. Further work is required to elucidate the optimal processing conditions for preparation of homogeneous, anhydrous, oxygen-free salt samples for testing. Of particular interest is the identification of temperatures/times which achieve homogeneity whilst minimising salt volatilisation. This may also necessitate further investment in laboratory-scale salt-handling facilities, such as dry gloveboxes containing milling, sectioning, grinding, and polishing equipment, and atmosphere-controlled furnaces.

The concentration of REEs at the bottom of the electrorefining samples and observation of REE-rich residues left after volatilisation are of interest for further investigation. The former may suggest that density of other effects could potentially be exploited to increase separation by utilising a vertical, rather than horizontal, zone-refining technique. The latter observation could indicate preferential volatilisation of the LKE over the REE chlorides, potentially opening up a new route for processing of this waste. Further work is required to investigate these potential opportunities further.

## Figures and Tables

**Table 1:** Simulant pyroprocessing salt waste compositions.

Component (wt.%)	Electroreducer Waste (No-Cs)	Electroreducer Waste (Full-Cs)	Electrorefiner Waste
LiCl	84.15	84.15	39.60
KCl	-	-	50.40
Li_2_O	0.85	0.85	-
BaCl_2_	9.72	4.92	-
CsCl	-	7.33	-
RbCl	1.40	0.73	-
SrCl_2_	3.88	2.02	-
YCl_3_	-	-	0.50
LaCl_3_	-	-	1.20
CeCl_3_	-	-	2.30
PrCl_3_	-	-	1.10
NdCl_3_	-	-	3.90
SmCl_3_	-	-	1.00

**Table 2:** Fit parameters for tube furnace temperature profiles.

	400 °C	450 °C	500 °C	550 °C	600 °C	650 °C	700 °C
a	−0.107	−0.055	−0.094	−0.077	−0.007	−0.176	−0.104
b	−2.283	−4.4049	−3.862	−4.409	−6.396	−3.738	−4.235
c	353.79	398.33	449.10	498.46	552.29	595.50	643.80

**Table 3:** Zone-refining parameters for electrorefining simulant samples.

Sample	Target Set Temp. (°C)	Est. Temp. at Inner End (°C)	Est. Temp. at Outer End (°C)	Set Cooling Rate (°C/min)	Est. Freezing-Front Vel., V (cm/s)
LKE-FSa	550	474	410	0.5	0.0014
LKE-FSb	550	474	410	0.1	0.0003
LKE-FSc	550	474	410	0.4	0.0011
LKE-FSd	550	474	410	0.3	0.0008
LKE-FSe	550	474	410	0.5	0.0014
LKE-FSf	550	474	410	0.5	0.0014

**Table 4:** Zone-refining parameters for electroreducing simulant samples.

Sample	Target Set Temp. (°C)	Est. Temp. at Inner End (°C)	Est. Temp. at Outer End (°C)	Set Cooling Rate (°C/min)	Est. Freezing-Front Vel., V (cm/s)
L-FSa	770	690	621	0.5	0.0012
L-FSb	770	690	621	0.5	0.0012

**Table 5:** Calculated DF_i_ values for zone refining of electrorefining simulant samples, X_R_ = 0.8.

	LKE-FSa	LKE-FSc	LKE-FSd	LKE-FSe	LKE-FSf
*DF_Nd_*	2.86	2.08	2.18	1.29	1.47
*DF_La_*	1.52	1.56	1.26	1.12	1.96
*DF_Ce_*	1.13	1.39	-	-	1.82
*DF_Y_*	1.23	1.73	1.56	1.08	1.15
*DF_Sm_*	1.85	1.65	2.45	1.25	1.31
*DF_Pr_*	1.81	1.72	1.81	1.24	1.37

**Table 6:** Calculated DF_i_ values for zone refining of electrorefining simulant samples, X_R_ = 0.9.

	LKE-FSa	LKE-FSc	LKE-FSd	LKE-FSe	LKE-FSf
*DF_Nd_*	3.60	2.47	2.61	1.38	1.62
*DF_La_*	1.69	1.75	1.34	1.16	2.30
*DF_Ce_*	1.17	1.52	-	-	2.10
*DF_Y_*	1.29	1.98	1.75	1.11	1.20
*DF_Sm_*	2.15	1.87	3.01	1.32	1.41
*DF_Pr_*	2.09	1.97	2.09	1.31	1.49

**Table 7:** Estimated electrorefining refined salt composition, X_R_ = 0.8.

wt.%	LKE-FSa	LKE-FSc	LKE-FSd	LKE-FSe	LKE-FSf
LiCl	40.29	40.17	40.14	39.75	40.04
KCl	51.27	51.13	51.09	50.60	50.96
YCl_3_	0.48	0.44	0.45	0.49	0.49
LaCl_3_	1.09	1.08	1.14	1.17	1.01
CeCl_3_	2.24	2.13	2.30	2.30	1.98
PrCl_3_	0.95	0.96	0.95	1.05	1.02
NdCl_3_	2.84	3.21	3.15	3.68	3.56
SmCl_3_	0.85	0.89	0.77	0.95	0.94
Total	100.00	100.00	100.00	100.00	100.00
Total RECl_3_	8.44	8.70	8.76	9.65	9.00

**Table 8:** Estimated electrorefining waste salt composition, X_R_ = 0.8.

wt.%	LKE-FSa	LKE-FSc	LKE-FSd	LKE-FSe	LKE-FSf
LiCl	36.88	37.32	37.43	38.98	37.84
KCl	46.93	47.49	47.64	49.61	48.16
YCl_3_	0.59	0.75	0.70	0.53	0.56
LaCl_3_	1.65	1.68	1.44	1.32	1.97
CeCl_3_	2.54	2.97	2.30	2.30	3.59
PrCl_3_	1.71	1.65	1.71	1.30	1.40
NdCl_3_	8.11	6.67	6.87	4.77	5.24
SmCl_3_	1.58	1.46	1.90	1.19	1.24
Total	100.00	100.00	100.00	100.00	100.00
Total RECl_3_	16.19	15.19	14.92	11.40	14.00

**Table 9:** Estimated electrorefining refined salt composition, X_R_ = 0.9.

wt.%	LKE-FSa	LKE-FSc	LKE-FSd	LKE-FSe	LKE-FSf
LiCl	40.11	40.00	39.99	39.70	39.91
KCl	51.05	50.91	50.90	50.53	50.79
YCl_3_	0.49	0.46	0.47	0.49	0.49
LaCl_3_	1.12	1.12	1.16	1.18	1.06
CeCl_3_	2.26	2.19	2.30	2.30	2.07
PrCl_3_	0.99	1.00	0.99	1.07	1.05
NdCl_3_	3.09	3.40	3.36	3.76	3.67
SmCl_3_	0.90	0.92	0.83	0.97	0.96
Total	100.00	100.00	100.00	100.00	100.00
Total RECl_3_	8.85	9.08	9.11	9.77	9.30

**Table 10:** Estimated electrorefining waste salt composition, X_R_ = 0.9.

wt.%	LKE-FSa	LKE-FSc	LKE-FSd	LKE-FSe	LKE-FSf
LiCl	35.07	35.97	36.08	38.68	36.85
KCl	44.63	45.78	45.91	49.23	46.90
YCl_3_	0.63	0.90	0.81	0.55	0.59
LaCl_3_	1.90	1.95	1.55	1.37	2.44
CeCl_3_	2.65	3.32	2.30	2.30	4.35
PrCl_3_	2.07	1.97	2.07	1.40	1.56
NdCl_3_	11.12	8.40	8.77	5.20	5.95
SmCl_3_	1.93	1.72	2.50	1.28	1.35
Total	100.00	100.00	100.00	100.00	100.00
Total RECl_3_	20.30	18.25	18.01	12.09	16.25

**Figure A2:**
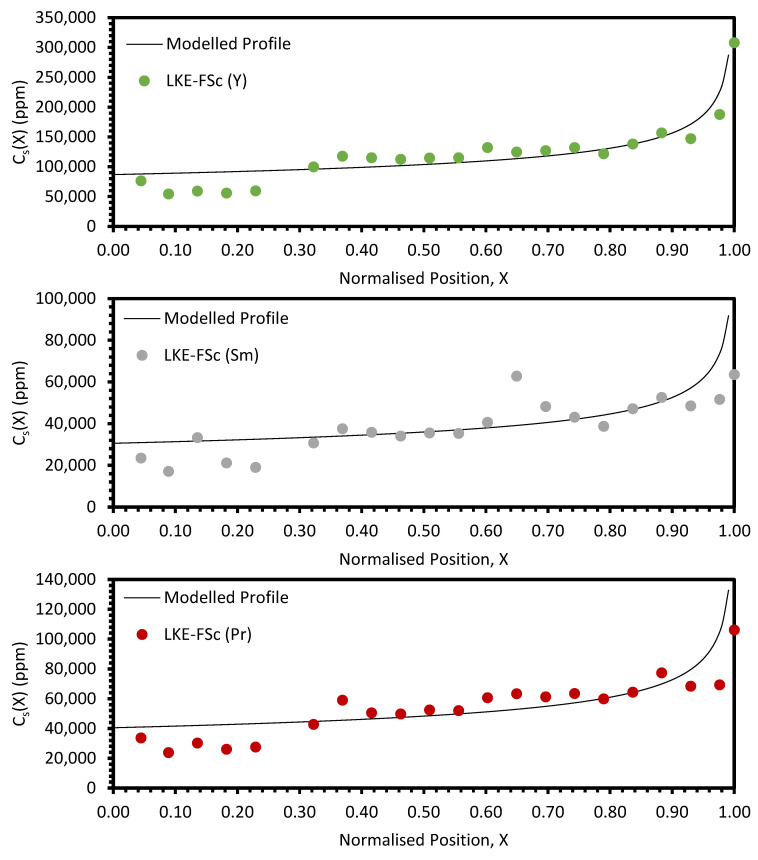
Experimental distribution profiles for Y, Sm, and Pr for the LKE-FSc sample compared to modelled profiles.

## Data Availability

Data are contained within the article.

## Appendix A

**Table A1:** Details of reagents purchased and used in this work.

Chemical	Supplier	Purity (%)	Notes
LiCl	BE	99	Anhydrous
KCl	BE	99.5	Anhydrous
SrCl_2_·6H_2_O	BE	98	Dehydrated at 350 °C for 60 h before use
BaCl_2_·2H_2_O	BE	99	Dehydrated at 350 °C for 60 h before use
YCl_3_	AA	99.9	
LaCl_3_	AA	99.9	
CeCl_3_	AA	99.5	
NdCl_3_	AA	99.9	
PrCl_3_	AA	99.99	
SmCl_3_	AA	99.5	
RbCl	AA	99.8	
CsCl	AA	99.9	

**Table A2:** Experimental details for initial tests of zone-refining apparatus.

Sample	Tracer	Melting Temp (°C)	Melting Duration (h)	Z-R Max Temp (°C)	Z-R Max Temp Hold (h)	Z-R Cooling Rate (°C/min)	Sample End Position (cm)
LKE-Ba2	2 wt.% BaCl_2_	N/A	N/A	1000	0.5	1.0	5.0
LKE-Ba2_b	2 wt.% BaCl_2_	400	2	500	2	1.0	5.0
LKE-Ba2_c	2 wt.% BaCl_2_	500	4	500	1	0.5	5.0
LKE-Ba5	5 wt.% BaCl_2_	500	4	500	2	0.4	5.0
LKE-Cs5	5 wt.% CsCl	500	8	500	2	0.2	5.0
LKE-Cs5_b	5 wt.% CsCl	500	8	500	8	0.2	5.0

**Table A3:** Measured furnace temperatures at horizontal position 0.0 cm.

Set Temperature (°C)	Measured Temperature (°C)
400	353
450	385
500	446
550	495
600	545
650	593
700	642

**Table A4:** Average R^2^ values for fitting of Equation (10) to electrorefining simulant data.

Sample	R^2^ (Nd)	R^2^ (Ce)	R^2^ (La)	R^2^ (Y)	R^2^ (Sm)	R^2^ (Pr)
LKE-FSa	0.004	0.011	0.002	0.020	0.036	0.011
LKE-FSc	0.051	0.140	0.040	0.039	0.056	0.046
LKE-FSd	0.036	-	0.012	0.024	0.043	0.032
LKE-FSe	0.029	-	0.023	0.012	0.027	0.023
LKE-FSf	0.019	0.072	0.039	0.010	0.033	0.018

**Table A5:** Average R^2^ values for fitting of Equation (10) to electroreducing simulant data.

Sample	R^2^ (Ba)	R^2^ (Sr)	R^2^ (Cs)	R^2^ (Rb)
L-FSa	0.077	0.083	0.115	0.110
L-FSb	0.057	0.057	0.071	0.065

**Table A6:** Model parameters of LKE-FSa experimental profiles.

Element	*k*	*V* (cm/s)	*δ* (cm)	*D* (cm^2^/s)	*k_eff_*
Nd	0.527	0.0014	0.001	2.40 × 10^−5^	0.542
La	0.778	0.0014	0.001	1.01 × 10^−5^	0.801
Ce	0.932	0.0014	0.001	1.28 × 10^−5^	0.938
Y	0.897	0.0014	0.001	3.00 × 10^−5^	0.901
Sm	0.690	0.0014	0.001	1.23 × 10^−5^	0.714
Pr	0.709	0.0014	0.001	2.00 × 10^−5^	0.724

**Table A7:** Model parameters of LKE-FSc experimental profiles.

Element	*k*	*V* (cm/s)	*δ* (cm)	*D* (cm^2^/s)	*k_eff_*
Nd	0.656	0.0011	0.001	2.40 × 10^−5^	0.666
La	0.770	0.0011	0.001	1.01 × 10^−5^	0.789
Ce	0.829	0.0011	0.001	1.28 × 10^−5^	0.841
Y	0.73	0.0011	0.001	3.00 × 10^−5^	0.737
Sm	0.84	0.0011	0.001	1.23 × 10^−5^	0.852
Pr	0.77	0.0011	0.001	2.00 × 10^−5^	0.780

**Table A8:** Model parameters of LKE-FSd experimental profiles.

Element	*k*	*V* (cm/s)	*δ* (cm)	*D* (cm^2^/s)	*k_eff_*
Nd	0.639	0.0008	0.001	2.40 × 10^−5^	0.647
La	0.880	0.0008	0.001	1.01 × 10^−5^	0.888
Ce	-	-	-	-	-
Y	0.776	0.0008	0.001	3.00 × 10^−5^	0.789
Sm	0.580	0.0008	0.001	1.23 × 10^−5^	0.600
Pr	0.708	0.0008	0.001	2.00 × 10^−5^	0.724

**Table A9:** Model parameters of LKE-FSe experimental profiles.

Element	*k*	*V* (cm/s)	*δ* (cm)	*D* (cm^2^/s)	*k_eff_*
Nd	0.868	0.0014	0.001	2.40 × 10^−5^	0.875
La	0.935	0.0014	0.001	1.01 × 10^−5^	0.943
Ce	-	-	-	-	-
Y	0.959	0.0014	0.001	3.00 × 10^−5^	0.961
Sm	0.879	0.0014	0.001	1.23 × 10^−5^	0.893
Pr	0.890	0.0014	0.001	2.00 × 10^−5^	0.896

**Table A10:** Model parameters of LKE-FSf experimental profiles.

Element	*k*	*V* (cm/s)	*δ* (cm)	*D* (cm^2^/s)	*k_eff_*
Nd	0.816	0.0014	0.001	2.40 × 10^−5^	0.825
La	0.661	0.0014	0.001	1.01 × 10^−5^	0.691
Ce	0.701	0.0014	0.001	1.28 × 10^−5^	0.723
Y	0.920	0.0014	0.001	3.00 × 10^−5^	0.930
Sm	0.851	0.0014	0.001	1.23 × 10^−5^	0.868
Pr	0.829	0.0014	0.001	2.00 × 10^−5^	0.848
